# Cortical responses correlate with speech performance in pre-lingually deaf cochlear implant children

**DOI:** 10.3389/fnins.2023.1126813

**Published:** 2023-06-02

**Authors:** Xiao-Qing Zhou, Qing-Ling Zhang, Xin Xi, Ming-Rong Leng, Hao Liu, Shu Liu, Ting Zhang, Wei Yuan

**Affiliations:** ^1^Department of Otolaryngology, Chongqing Medical University, Chongqing, China; ^2^Chongqing Institute of Green and Intelligent Technology, Chinese Academy of Sciences, Chongqing, China; ^3^Chongqing School, University of Chinese Academy of Sciences, Chongqing, China; ^4^Department of Otolaryngology, Chongqing General Hospital, Chongqing, China; ^5^Department of Otolaryngology Head and Neck Surgery, Chinese PLA General Hospital, Beijing, China; ^6^Chongqing Integrated Service Center for Disabled Persons, Chongqing, China

**Keywords:** cochlear implant, cortical activation, cross-modal reorganization, speech understanding, functional near-infrared spectroscopy

## Abstract

**Introduction:**

Cochlear implantation is currently the most successful intervention for severe-to-profound sensorineural hearing loss, particularly in deaf infants and children. Nonetheless, there remains a significant degree of variability in the outcomes of CI post-implantation. The purpose of this study was to understand the cortical correlates of the variability in speech outcomes with a cochlear implant in pre-lingually deaf children using functional near-infrared spectroscopy (fNIRS), an emerging brain-imaging technique.

**Methods:**

In this experiment, cortical activities when processing visual speech and two levels of auditory speech, including auditory speech in quiet and in noise with signal-to-noise ratios of 10 dB, were examined in 38 CI recipients with pre-lingual deafness and 36 normally hearing children whose age and sex matched CI users. The HOPE corpus (a corpus of Mandarin sentences) was used to generate speech stimuli. The regions of interest (ROIs) for the fNIRS measurements were fronto-temporal-parietal networks involved in language processing, including bilateral superior temporal gyrus, left inferior frontal gyrus, and bilateral inferior parietal lobes.

**Results:**

The fNIRS results confirmed and extended findings previously reported in the neuroimaging literature. Firstly, cortical responses of superior temporal gyrus to both auditory and visual speech in CI users were directly correlated to auditory speech perception scores, with the strongest positive association between the levels of cross-modal reorganization and CI outcome. Secondly, compared to NH controls, CI users, particularly those with good speech perception, showed larger cortical activation in the left inferior frontal gyrus in response to all speech stimuli used in the experiment.

**Discussion:**

In conclusion, cross-modal activation to visual speech in the auditory cortex of pre-lingually deaf CI children may be at least one of the neural bases of highly variable CI performance due to its beneficial effects for speech understanding, thus supporting the prediction and assessment of CI outcomes in clinic. Additionally, cortical activation of the left inferior frontal gyrus may be a cortical marker for effortful listening.

## 1. Introduction

A cochlear implant (CI) is currently the only FDA-approved biomedical device that can restore hearing for the majority of individuals with severe-to-profound sensorineural hearing loss (SNHL). Despite the fact that speech restoration with a CI has generally been successful in cases of deaf children (Nikolopoulos et al., 2004; Wiley et al., 2005; Sharma and Dorman, 2006), there is still a great deal of variability in CI post-implantation results (Niparko et al., 2010; Geers et al., 2011), particularly when listening to speech amid background noise (Saksida et al., 2022). It is unknown why some implanted children experience poor speech perception following implantation. Several factors such as rehabilitative communication strategy, age at onset of hearing loss, duration of deafness, age at cochlear implantation, experience of hearing aid use, and duration of CI experience contribute to speech perception outcomes, but huge variance in auditory skill development remains unexplained in children with CIs (Zeng, 2004; Tomblin et al., 2005; Lin et al., 2008; Niparko et al., 2010; Tobey et al., 2013). Therefore, seeking an accurate predictor or measure is extremely important to assist clinicians in better anticipating clinical outcomes, tracking subsequent adaptation to the restored auditory input, ultimately aiding clinical settings, supporting adequate and timely rehabilitation, and implementing interventions.

It has been proposed that auditory-to-visual cross-modal plasticity driven by hearing loss may play a significant role in understanding and predicting the potential benefits of post-lingually adult CI users (Doucet et al., 2006; Sandmann et al., 2012; Strelnikov et al., 2013; Song et al., 2015; Anderson et al., 2017; Fullerton et al., 2022). This neuroplasticity could provide adaptive benefits after hearing deprivation by enhancing the abilities of non-auditory skills, such as superior visual speechreading skills (Rouger et al., 2007); on the other hand, it was also demonstrated to correlate with behavioral measures of speech performance (Strelnikov et al., 2013; Anderson et al., 2017; Fullerton et al., 2022). Those adult CI research literature showed that cross-modal plasticity may be another factor affecting speech perception outcomes in cochlear implanted children. However, it remains unclear how such cortical reorganization of brain regions might influence hearing restoration in pre-lingually deaf children after implantation.

In children who are pre-lingually deaf, deprivation of auditory input during sensitive periods impedes the normal development of central auditory pathways and is associated with heightened sensitivity to visual stimuli observed in auditory brain regions. This cross-modal plasticity was believed to be harmful to CI outcomes because it prevented the auditory cortical areas from processing newly introduced auditory stimuli (Lee et al., 2001; Giraud and Lee, 2007; Lee et al., 2007). The reason why cochlear implantation should be performed as early as possible was probably because early implantation could prevent cross-modal takeover of auditory regions (Lee et al., 2007). However, in recent years, this view was thought to be overly simplistic (Heimler et al., 2014). Instead, the activation of auditory cortical areas by visual speech may not hinder the recovery of the auditory sense following implantation but may help preserve important language networks, which may improve CI results (Lyness et al., 2013; Mushtaq et al., 2020). Therefore, it is necessary to explore the relationship between cortical cross-modal activation and speech outcomes in CI children further. Functional near-infrared spectroscopy (fNIRS), an emerging brain-imaging technique, is considered to be one of the most suitable means of neuroscience research for people with hearing loss or hearing devices, due to its advantages of being CI compatible, noninvasive, quiet, safe for repeated use, unrestrictive and tolerant of movement artifact (Hoshi, 2003; Kiguchi et al., 2007; Dieler et al., 2012). Evidence related to using fNIRS to explore cortical plasticity in CI adults with post-lingual deafness has demonstrated its validity and feasibility (Olds et al., 2016; Anderson et al., 2017; Zhou et al., 2018). The purpose of this study was to apply fNIRS to examine the influence of cross-modal plasticity in defined regions of interest (ROIs) on speech understanding in a large sample of pre-lingually deaf CI children with a more diverse range of speech abilities.

Previous neuroimaging studies examining visual takeover of auditory regions in CI children often used low-level visual stimuli such as checkerboards (Corina et al., 2017) and pictures (Liang et al., 2017). Compared to those visual non-speech materials, speech stimuli contain more information and are more representative in terms of communication and language. In the case of post-lingually deaf CI adults, cross-modal activation of auditory cortex by visual speech was demonstrated to be beneficial for speech performance with a CI (Anderson et al., 2017; Fullerton et al., 2022). Unlike post-lingually acquired deafness, pre-lingually deaf children who did not have an experience of using visual cues when listening to speech may show different results between response of auditory cortex to visual speech and speech understanding after implantation. Additionally, it has been controversial whether visual speech (lip-reading) should be used in current CI rehabilitation strategies due to the correlation between cross-modal plasticity and CI outcomes. Therefore, visual speech (lip-reading) was used as the visual stimulus in this study. Bilateral superior temporal gyrus (STG, Brodmann area 22) and left inferior frontal gyrus (LIFG, Brodmann areas 44 and 45), as well as bilateral inferior parietal lobes (IPL, Brodmann areas 39 and 40), were defined as ROIs beforehand because activation of fronto-temporal–parietal regions, particularly the network dominated by STG, was involved in speech comprehension in CI recipients (Lee et al., 2005; Anderson et al., 2017; Zhou et al., 2018) and normally-hearing (NH) subjects (Wijayasiri et al., 2017; Defenderfer et al., 2021; Lawrence et al., 2021). In brief, increased visual processing in STG is associated with variable auditory performance with a CI (Strelnikov et al., 2013; Chen et al., 2016; Anderson et al., 2017; Zhou et al., 2018; Anderson et al., 2019; Mushtaq et al., 2020), and either LIFG (Wong et al., 2008; Obleser and Kotz, 2010) or IPL (Lawrence et al., 2018; Mushtaq et al., 2021) is crucial for improving speech recognition under challenging listening situations, such as listening to speech in background noise or recovering meaning from degraded speech.

The aims of the present study were to (i) examine the impacts of bilateral STG activation to visual speech on speech understanding in children with CIs (and a group of NH controls); (ii) explore underlying mechanisms of the relationship between cross-modal brain plasticity and speech performance after implantation; and (iii) measure activities in LIFG and IPL during listening to speech with two levels. To achieve these aims, we implemented a fNIRS experiment using a block design and examined cortical responses in defined ROIs during three conditions: auditory speech in quiet (SIQ), auditory speech in noise (SIN), and visual speech. We hypothesized that: (i) pediatric CI users would elicit stronger cross-modal responses to visual speech in auditory brain regions compared with NH controls because of early auditory deprivation; (ii) NH listeners would elicit stronger responses to auditory speech than CI users to reflect retained auditory processing specialization of the auditory cortex; and (iii) the amplitude of LIFG and IPL activation would vary according to speech condition. To our knowledge, this is the first fNIRS study to describe neural activation of fronto-temporal–parietal networks in a representative sample of pediatric CI recipients with pre-lingual deafness.

## 2. Materials and methods

### 2.1. Participants

The study protocol was approved by Chongqing General Hospital and conformed to the declaration of Helsinki. Before taking part, all participants’ accompanying guardians signed informed consent forms, and subjects were also asked to verbally assent to attend. CI users were contacted through the Chongqing Integrated Service Center for Disabled Persons. NH controls were school-age students or acquaintances of the project’s researchers, who were recruited through word-of-mouth or online advertisements. Ages between 6 and 12 years old, native Mandarin speakers, healthy, and self-reported or parent-reported normal or corrected-to-normal vision were common inclusion criteria across both groups. Exclusion criteria were any known language, cognitive, or motor disorder; a history of brain injury; and any active external or middle ear disease. Additionally, to eliminate discrepancies in handedness, the Edinburgh Handedness Inventory (Oldfield, 1971) was used to confirm that each individual was right-handed.

In order to rule out the side of implantation as a contributing factor in the analysis, only CI users with a right-ear implant were engaged. All of the participants in the CI group were pre-lingually deaf children who had used their right-ear implants for more than 1 year. CI participants were questioned about their deafness, including the etiology of deafness, age at onset and duration of deafness, history of hearing aid use, age at CI activation and duration of CI use. Briefly, all children received hearing screening at birth and had no genetic damage to organs other than the ear. In patients with congenital or early-onset deafness (later than at birth) caused by meningitis (three subjects), auditory neuropathy (two subjects), congenital malformation of inner ear (one subjects) and enlarged vestibular aqueducts (four subjects). Only a small percentage of children underwent genetic screening due to family financial reasons, and two of them had unspecified genetic causes of deafness. The etiology of hearing loss was unknown for 26 subjects. Twenty-four of the children had used hearing aids prior to CI, while the remaining 14 had not. However, the duration of hearing aid use was extremely varied, ranging from complete absence to continuous bilateral use. Table 1 presents the details regarding CI participants.

**Table 1:** Demographic characteristics of CI users, including speech understanding scores.

Subject ID	Gender	Age (years)	Onset (months)	Duration (months)	HA history	CI age (years)	CI side	CI duration (months)	MSP (quiet, %)	MSP (SNR10dB, %)
CI_01	Female	6.83	18	8	Yes	2.14	B	57	80	65.7
CI_02	Female	7.84	12	28	Yes	3.28	R	55	21.4	10.7
CI_03	Male	6.56	At birth	14	Yes	1.17	R	66	94.3	87.1
CI_04	Female	6.01	At birth	18	Yes	1.47	R	55	58.6	7.1
CI_05	Male	7.23	10	52	Yes	5.10	R	26	71.4	51.4
CI_06	Male	6.30	At birth	24	Yes	1.94	R	53	91.4	87.1
CI_07	Female	7.91	28	33	Yes	5.01	R	35	31.4	0
CI_08	Male	6.96	At birth	33	Yes	2.73	R	52	92.9	80
CI_09	Male	6.13	12	7	Yes	1.55	R	56	40	4.3
CI_10	Female	6.55	At birth	43	No	3.56	R	36	94.3	78.6
CI_11	Male	6.13	18	15	No	2.72	R	42	88.6	54.3
CI_12	Male	7.15	14	48	No	5.12	R	25	60	40
CI_13	Male	8.15	12	36	No	3.92	R	52	25.7	7.1
CI_14	Male	8.19	18	26	No	3.66	R	55	88.6	84.3
CI_15	Male	6.57	At birth	19	Yes	1.59	R	61	38.6	0
CI_16	Male	7.98	At birth	61	No	5.03	R	36	17.1	0
CI_17	Male	7.28	At birth	30	No	2.44	R	59	75.7	75
CI_18	Male	7.88	At birth	54	Yes	4.42	R	42	71.4	65.7
CI_19	Female	6.22	At birth	20	Yes	1.64	R	56	50	8.6
CI_20	Female	6.30	At birth	12	No	1.00	R	64	94.3	75.7
CI_21	Male	6.77	At birth	38	Yes	3.15	R	44	94.3	77.1
CI_22	Female	6.30	17	3	No	1.65	B	57	95.7	82.9
CI_23	Male	6.36	19	11	Yes	2.51	R	47	72.9	72.9
CI_24	Female	6.02	12	4	No	1.38	B	57	77.1	75.7
CI_25	Male	6.46	18	34	Yes	4.33	R	26	15.7	12.3
CI_26	Male	6.07	18	14	Yes	2.67	R	41	24.3	0
CI_27	Male	6.70	18	28	Yes	3.80	R	35	47.1	42.9
CI_28	Male	8.16	24	16	Yes	3.32	R	59	90	75.7
CI_29	Male	6.95	18	12	Yes	2.52	R	54	95.7	87.1
CI_30	Male	6.84	12	24	Yes	2.94	R	47	45.7	35.7
CI_31	Male	6.24	16	7	Yes	1.94	R	52	74.3	63
CI_32	Male	6.89	35	8	No	3.56	R	41	67.1	67.1
CI_33	Male	6.69	24	21	No	3.72	R	36	84.3	74.3
CI_34	Female	6.74	34	10	Yes	3.67	R	37	75.7	62.9
CI_35	Female	6.02	At birth	31	No	2.59	R	42	72.9	65.7
CI_36	Male	6.15	12	14	No	2.19	R	48	78.6	51.4
CI_37	Male	7.46	12	20	Yes	2.66	R	58	64.3	51.4
CI_38	Male	7.84	12	21	Yes	2.73	B	62	71.4	35.7

Age, natural age (years); Onset, age at onset of bilateral hearing loss (months); Duration, duration of bilateral hearing loss (months); CI age, age at cochlear implantation (years); CI side, side of cochlear implantation; B, bilateral; R, right; CI duration, duration of CI use since activation of CI device in right side (months); HA history, Experience of hearing aid use before implantation; MSP, Mandarin Speech Perception; SNR, signal-to-noise ratio.

The NH listeners recruited for this study were age and gender matched with CI recipients. These children were healthy and had pure-tone air conduction thresholds of ≤20 dB SPL at 0.5, 1, 2, and 4 kHz in both ears.

Forty-three pre-lingually deaf children with CI and 41 NH subjects participated in this study. Two CI children withdrew from the fNIRS examination because they could not tolerate the optodes on their heads. Moreover, three CI children and five NH children were excluded due to excessively poor channel quality. Eventually, available data was obtained from 38 pre-lingually deaf CI children (mean age 6.86 ± 0.70 years, range 6.01–8.19 years, 11 females) and 36 control subjects (mean age 7.04 ± 0.89 years, range 6.05–8.87 years, 14 females) participated in the study. There were no significant differences in age and gender between the two groups (both *p* > 0.05). This sample size was determined using data from earlier fNIRS investigations with CI recipients utilizing similar stimuli (Anderson et al., 2017; Zhou et al., 2018; Anderson et al., 2019; Mushtaq et al., 2020). Along with it, the Hiskey-Nebraska Test of Learning Aptitude (H-HTLA) was used to assess intelligence, and none of the subjects were intellectually disabled. All participants were fluent in Mandarin Chinese similar to the Chongqing dialect.

### 2.2. Speech understanding test

Prior to neuroimaging testing, the auditory speech perception abilities of all participants were measured in a soundproof room in which the background noise level was less than 30 dBA. A GSI free-field loudspeaker was used to deliver auditory stimuli, and the speech processor program in the CI user was configured in clinical settings throughout the test. CI users who had an implant or a hearing aid in the left ear were instructed to remove the device. Open-set disyllabic words from Mandarin Speech Perception (MSP) material (Zhu et al., 2012) were used to obtain a measure of speech perception. This material consisted of 10 standardized lists, each including 35 words recited by a female talker. MSP words were delivered to participants at a presentation level of 65 dBA. To prevent ceiling effects, these words were presented both in quiet and in steady, speech-shaped noise, with signal-to-noise ratios (SNRs) of 10 dB. For each condition, a list was randomly selected out of a group of 10 lists, each list with 35 words, and a disyllabic word was randomly selected from the list. Following each word presentation, the participant was told to pay close attention to the words and try their best to repeat back every word. A licensed audiologist scored participants’ responses to MSP words according to the proportion of words they correctly identified. No lists were repeated within test subjects.

At the start of the speech perception test, all participants completed a short practice that was performed simply and not scored to ensure that they all understood the procedure of behavioral measures. Notably, no participant received the same word more than once, and none of the subjects received any feedback at any point in the experiment.

### 2.3. fNIRS stimuli

The HOPE corpus, which was used to generate speech stimuli during the acquisition of fNIRS measurements, is a corpus of Mandarin sentences with paired babble noises that are similar to Bamford-Kowal-Bench (BKB) sentences (Xi et al., 2012). This material comprised digital audiovisual recordings of 160 sentences that were transcribed in a sound-attenuating test booth at the Chinese PLA General Hospital, Beijing, and were male-spoken, phonemically-balanced. There were between six and eight words in each sentence, with three or four of those being defined as keywords. An illustration of a sentence with keywords underlined is: “她看见一只兔子/She recognized a rabbit./” We selected 63 sentences from the material to use for testing, so there were seven sentences in each of the nine blocks. To draw the participant’s attention, a sentence including an animal was contained in every block. Except for specific sentences involving animals, which were subsequently distributed at random to each block, all sentences were chosen randomly from the corpus.

The experiment included a visual and an auditory session. For the auditory session, we designed two listening conditions: SIQ and SIN, where the auditory speech cues were presented but the visual speech cues were not shown. First, sentences were digitally isolated from their respective lists into 4-s trials using Adobe Audition editing software. Subsequently, in SIQ trials, babble noise in the right channel was removed, and only male-spoken Mandarin sentences in the left channel were retained. SIN trials were created by first modifying the 4-s noise in the right channel to reflect a total root-mean-square (RMS) amplitude value of 10 dB lower than the total RMS of the individual sentence to generate a specific SNR (+10 dB). Next, the babble noise and Mandarin sentences were mixed in the left channel. For the visual session, we adopted visual speech (i.e., lip-reading), where the visual speech cues of the recording were shown but the auditory speech cues were muted. The visual stimuli consisted of lip-reading of HOPE sentences and were also edited from their respective lists into 4-s trials using Adobe After Effects software according to the auditory stimuli. The background of the two auditory speech conditions was uniform, and the talker’s mouth was replaced with a fixation cross. Only this uniform background and fixation cross were used during rest intervals.

### 2.4. fNIRS paradigm

**Figure 1:**
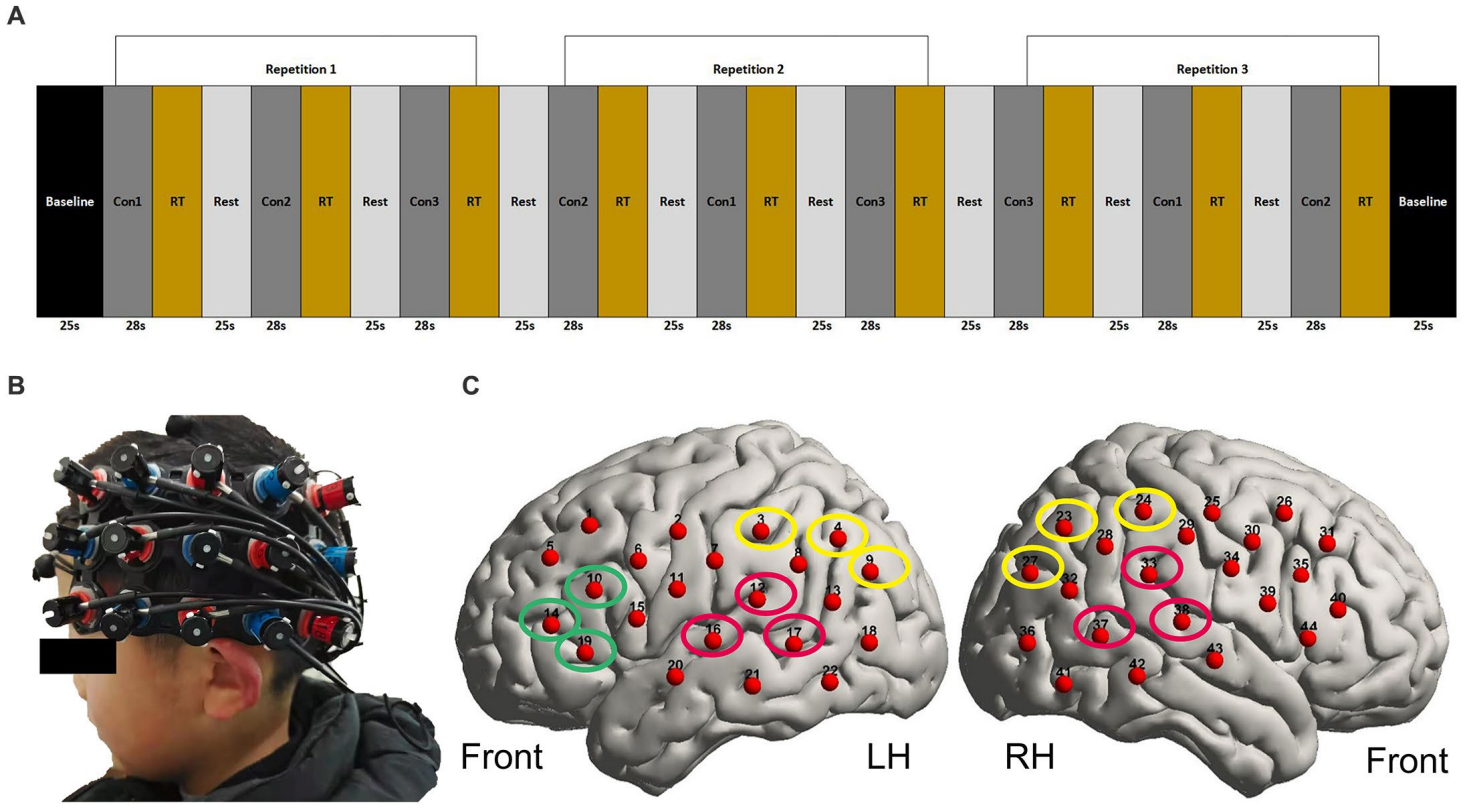
fNIRS paradigm and the localization of optodes. **(A)** Illustration of three repetitions of each stimulus type in pseudorandom order. Con1 represents SIQ (28 s), Con2 represents visual speech stimuli (28 s), Con3 represents SIN (28 s), and RT represents the response time in which a behavioral task was presented. The baseline and rest periods lasted 25 s each. **(B)** A photograph of the optode array holder placed on the head of one of the participants. The red and blue color coding on the holder indicates the locations of emitters and detectors, respectively. **(C)** fNIRS measurement channel locations on the brain cortex using a 3D digitizer. The channels outlined in red form bilateral STG. The channels outlined in green form LIFG. The channels outlined in yellow form bilateral IPL. LH, left hemisphere; RH, right hemisphere.

The speech stimuli were presented in a pseudorandom block design, with a baseline of 25 s followed by 9 blocks of stimuli that alternated between SIQ, SIN, and visual speech stimuli (Figure 1A). A no-stimulus period (rest) with a duration of 25 s was incorporated between those blocks to allow the haemodynamic response produced by the stimulation block to return to a baseline level. Each block contained seven sentences, evenly spaced to fill a 28-s block duration. Participants were told to pay attention to the talker and make an effort to comprehend what the talker was saying throughout these blocks. For the visual condition, participants were instructed to fixate on the location of the talker’s mouth. For the auditory conditions and rest periods, participants were instructed to look at the centrally positioned fixation cross and to minimize saccades as much as possible. To maintain attention to the speech stimuli throughout the experiment, an attentional trial was presented after each of the blocks. Two alternative animal pictures were presented on either side of the fixation cross 0.5 s after the presentation of each block, in which one animal in the picture had appeared in the previous block and the other animal in the picture almost rhymed with the correct animal. Participants were required to select the animal picture that appeared in the immediately preceding sentences they had just heard by pressing one of two buttons. They had up to 6 s to respond; otherwise, the pictures would disappear. We used this task only to ensure that subjects could focus their attention during the neuroimaging test phase, but the behavioral task results were not included in the analysis.

Before fNIRS scanning, participants first completed a brief familiarization run to make sure they understood the experimental procedure. The familiarization blocks contained sentences that were different from those delivered during the fNIRS measurements and the behavioral assessment in order to prevent preexposure to the experimental stimuli. This practice task was redone several times if the subject made mistakes until the researcher confirmed that the participant understood the task completely. Notably, speech stimuli in speech understanding tests differed from those in the corpus, which helped to limit training effects within and across testing sessions.

### 2.5. fNIRS measurements

The experiment was performed in the same booth as the speech perception test, with lights out in the room while collecting data. Participants were situated comfortably at a distance of 75 cm from a computer (Thinkpad E480) display unit, which was utilized to present visual stimuli. Auditory stimuli were delivered through a GSI free-field speaker placed directly on the monitor at a presentation level where sound intensity was coordinated at 70 dB SPL (A-weighted) as measured by a sound level meter when the subjects were absent. Although ear inserts do improve the SNR for the delivery of auditory stimuli, sound field presentation was more effectively and accurately to represent “real-world” experience with spoken communication (Hervais-Adelman, 2012). Before the experiment, participants removed their hearing device in the left ear if they had one. The stimuli of the study were presented through the Eprime3.0 (Psychology Software Tools, Inc., Pittsburgh, PA, United States) tool. Brain activity was non-invasively measured using a Hitachi ETG-4100 (Hitachi Medical Corporation, Tokyo, Japan) optical topography system, which emitted infrared light at wavelengths of 695 and 830 nm and sampled at a rate of 10 Hz, as well as used frequency modulation to minimize crosstalk between channels and wavelengths (Scholkmann et al., 2014).

A pair 3 × 5 optode arrays were placed over the left and right temporal regions, aiming to mainly cover the bilateral STG, LIFG, and bilateral IPL. Together, these consisted of 16 sources and 14 detectors with a 3-cm fixed source-detector gap, resulting in 44 measurement channels (22 per hemisphere). As shown in Figure 1B, to standardize array placement across participants, the middle optode on the bottom row was positioned close to the preauricular point and the middle optode on the top row was pointed in the direction of point Cz according to the 10–20 system (Klem et al., 1999). Importantly, there was some variation in how the external CI processor was positioned among the participants in the CI group, so that the external CI processor sometimes interfered with probe placement. In such cases, we positioned the headset over the processor. While this prevented certain channels from scalp contact, the data acquisition of the remaining channels was usable. To improve optode-scalp contact, we carefully removed redundant hair from underneath optodes with a small plastic illuminated tool, modified the angle of the optodes, and ran the signal check program that was pre-installed in the ETG 4100. Until all of the accessible channels passed the signal test, we did not move on to the next phase. To further guarantee the consistency of optode placement, a reference picture was taken once the position of the array had been settled upon. During imaging, individuals were required to keep as still as possible and avoid unnecessary head movements to reduce motion artifacts in the fNIRS data. Prior to starting the neuroimaging task during data collection, participants received verbal and written instructions. The task was then started at the participant’s decision by pressing the spacebar on the keyboard. The onset and end of each stimulus were timed to match the beginning and finish of the incoming fNIRS data, and they were both recorded in an event file. Participants did not receive any feedback on their performance accuracy.

### 2.6. Processing of fNIRS data

#### 2.6.1. fNIRS data for cortical activation

The fNIRS recordings were imported into MATLAB (R2013A; The MathWorks) for further analysis using HOMER2 (Huppert et al., 2009) and NIRS-SPM (Jong et al., 2008) toolboxes together with custom scripts. Pre-processing of the data was performed using HOMER2 software, and the fNIRS response amplitude was quantified using NIRS_SPM software.

Before processing of the data, the task-unrelated time intervals were removed first. Following that, because poor optode-scalp contact can be a limiting factor impacting fNIRS data quality, the scalp coupling index (SCI) approach introduced by Pollonini et al. (2014) and visual inspection were used to exclude channels from which data were unacceptable in quality. In order to maintain as many channels as possible for further statistical analysis, we established a flexible threshold of SCI ≥ 0.202 and decided to just remove the worst 5% of channels from the overall dataset.

Processing of the data for the retained channels proceeded as follows:

- The raw intensity signals from each channel were converted to changes in optical density using the HOMER2 hmrIntensity2OD function (Huppert et al., 2009).
- A correction strategy was chosen to reduce signal contamination since children may exhibit motion/muscle artifacts. We first used spline interpolation approach (*p* = 0.99, frame size = 10 s) to remove large spikes and baseline shifts in the data (Scholkmann et al., 2010). Second, we used the HOMER2 package’s hmrMotionCorrectWavelet function (IQR = 0.7), which implements a condensed version of the algorithm proposed by Molavi and Dumont (2012). During experiments involving speech tasks, this function has been demonstrated to significantly reduce motion artifact (Cooper et al., 2012; Brigadoi et al., 2014). We did not include wavelet coefficients that were more than 0.7 times either the first or third quartile interquartile range. If the wavelet coefficients are normally distributed, this almost corresponds to the α = 0.1 threshold used in assessing motion artifact corrections for fNIRS methods (Lawrence et al., 2021).
- Following motion-artifact correction, recordings were bandpass filtered with cut-off frequencies of 0.01 and 0.5 Hz for the lower and upper thresholds to reduce the physiological noise sources in the data, such as high-frequency cardiac oscillations, low-frequency respiration, and blood pressure changes (Dewey and Hartley, 2015; Yucel et al., 2021).
- The optical density data were transformed into estimated changes in HbO and HbR concentrations using the modified Beer–Lambert law after motion-artifact correction (Huppert et al., 2009). We adopted a default value of 6 for the differential path-length factor at both wavelengths.
- An anti-correlation method (Yamada et al., 2012), which assumes that systemic noise-induced changes in HbO and HbR concentration are positively correlated but stimulus-related changes in HbO and HbR concentration tend to be negatively correlated, was used as the final stage of pre-processing to further reduce physiological interference. The HbO and HbR associated to the stimuli in channels were identified by maximizing the negative correlation between them (Cui et al., 2010).
- After completing the necessary pre-processing steps, we used the general linear model (GLM) approach to calculate the level of cortical activation (Schroeter et al., 2004). The stimulus time-course convolved with a canonical hemodynamic response function implemented in SPM 8 software (Wellcome Trust Centre for Neuroimaging, UCL, UK, 2009) together with its temporal and dispersion derivatives (Ho, 2012). Finally, we utilized the beta value to evaluate the impact of the stimulus on cortical response. The beta value was block averaged over three repetitions of each stimulus to obtain the mean hemodynamic response of each participant, channel, and stimulus condition. The estimated response amplitudes (ERAs) within each ROI were the mean beta values across the ROI measurement channels. Additionally, this study focused on HbO responses since they are more sensitive to changes in regional cerebral blood flow (Hoshi, 2007).

#### 2.6.2. fNIRS data for functional connectivity

The Homer2 toolbox was used to process the data for the functional connectivity analysis together with custom scripts (Scholkmann et al., 2014). Consistent with the pre-processing of the activation analysis, including exclusion of channel, artifact rejection, motion correction, bandpass filtering (0.009–0.1 Hz), the Modified Beer–Lambert Law, and estimation of the hemoglobin concentrations. We used a different filter range for functional connectivity as compared to the activation analysis. This is because previous research has shown high coherence in a low-frequency range (0.009–0.1 Hz) (Sasai et al., 2011). Then the hemoglobin concentrations were segmented into an epoch corresponding to the window in which the stimulus was shown and a response was generated (−5 to +30s). It has indicated that the HbO data exhibits more robust coherence patterns and connectivity than HbR data; consequently, connectivity analysis was carried out using HbO data (Wolf et al., 2011). The coherence between all channels was evaluated for each participant employing epoch data within the frequency range of 0.009–0.1 Hz (Yucel et al., 2021). The resulting coherence values indicate the degree of similarity in signals between channel pairs during the outlined time window. A value closer to 1 suggests a higher degree of similarity, while a value closer to 0 suggests greater independence of signals (Fullerton et al., 2022). Coherence values for the ROI channels (Figure 1C) were averaged to estimate task-related connectivity during speech processing. Specifically, connectivity included coherence values between 7 ROI pairs: LSTG and RSTG, LSTG and LIFG, LSTG and LIPL, LSTG and RIPL, RSTG and LIFG, RSTG and LIPL, and RSTG and RIPL.

### 2.7. Definition of ROI

ROIs were pre-selected for this study. The main *a priori* “auditory” ROI targeted superior temporal regions considering recent fNIRS research on cross-modal brain plasticity in CI users (Olds et al., 2016; Anderson et al., 2017; Zhou et al., 2018; Anderson et al., 2019; Mushtaq et al., 2020) and comprised symmetrical channels 12, 16, and 17 in the left hemisphere (LH) and channels 33, 37, and 38 in the right hemisphere (RH). A pair of secondary *a priori* ROIs targeted “LIFG” regions (including channels 10, 14, and 19 in the LH) and “bilateral IPL” regions (namely channels 3, 4, and 9 in the LH and channels 23, 24, and 27 in the RH), the selection of which was based on their potential influence on effortful listening (Wong et al., 2008; Obleser and Kotz, 2010; Adank, 2012; Wild et al., 2012; Lawrence et al., 2018, 2021). In order to estimate channel positions on the cortical surface, the optode placement was recorded using the Hitachi ETG-4100’s electromagnetic 3D Probe Positioning Unit, as illustrated in Figure 1C. First, the 3D digitizer system was used to record the positions of the optodes and anatomical surface landmarks (the left tragus, right tragus, nasion, inion, and Cz), which were then translated into MNI coordinates using MATLAB (R2013A; The MathWorks) with customized scripts. Finally, these coordinates were input into the NIRS-SPM toolbox to register fNIRS channels and project them to brain regions.

### 2.8. Statistical analysis

Both behavioral and fNIRS data were analyzed using IBM SPSS Statistics for Windows Version 25.0 software (IBM Corp., Armonk, New York). The reported *p*-values in all analyses were two-tailed, with a significance level set at *p* < 0.05 without any special instructions. Furthermore, we used the Bonferroni method to correct for multiple comparisons of *p*-values. Speech understanding was quantified as the percentage of words reported correctly (% correct). To make the data more suitable for statistical analysis, the rationalized arcsine transform was applied using SPSS 25 (Anderson et al., 2017). Subsequently, the transformed scores [rationalized arcsine units (RAUs)] were subjected to statistical analysis.

In each group, we employed two-tailed t-tests to evaluate cortical activation in a total of 44 measurement channels. Specifically, we contrasted each speech condition against a silent baseline and applied a false discovery rate (FDR) correction method (Benjamini and Hochberg, 1995) to adjust for multiple comparisons across all channels. To ensure high statistical rigor, we established an FDR-corrected threshold of *q* < 0.05 indicating statistical significance.

The cortical activation differences in each ROI were determined by analyzing the ERAs for the bilateral STG, bilateral IPL, and LIFG separately using three linear mixed models (LMMs). The first two LMMs included fixed effects of “group” (CI vs. NH or GCI vs. PCI), “stimulus type” (SIQ vs. SIN vs. visual condition), and “hemisphere” (LH vs. RH), with all two- and three-way interactions, as well as a random intercept for “participant.” When specifically examining the cortical activation differences in LIFG, the models included fixed effects of “group,” “stimulus type,” and “group-stimulus type,” along with a random intercept for “participant.” The task-related functional connectivity differences between groups in each ROI pair were determined by analyzing the coherence values for SIQ, SIN, and visual condition separately using three LMMs, including fixed effects of “group” (CI vs. NH), “ROI pair” (7 pairs of ROI), group×ROI pair interaction, and a random intercept for “participant.” Estimation of the model parameters was done through the restricted maximum likelihood (REML) approach. The *post hoc* Bonferroni’s test was used for multiple comparisons during follow-up analyses.

Bivariate correlation analysis was conducted to examine the association between activation levels (ERAs) or coherence values and speech perception scores (RAU). Specifically, the parametric statistic Pearson’s correlation coefficient (r) was used to estimate the direction and strength of the linear relationship. Since the age-at-onset, duration of deafness prior to implantation, age-at-implantation, and duration of CI use are known clinical factors influencing CI outcomes (Zeng, 2004; Tomblin et al., 2005; Green et al., 2007; Lin et al., 2008; Niparko et al., 2010; Lazard et al., 2012; Blamey et al., 2013; Tobey et al., 2013), correlation analysis was also conducted between these factors and speech performance with a CI. If there were some correlations, partial correlation analysis would be used to control the impacts of these factors.

## 3. Results

### 3.1. Behavioral results: speech performance

All NH children scored 100% on both speech understanding tests, with the exception of one child who scored 98.29% in quiet and 97.14% in noise. In contrast, the deaf children with CIs displayed a huge amount of variability in their performance on the behavioral tests. A summary of the percentage of correctly identified words in both parts of speech perception test by each CI user is shown in Table 1. The scores ranged from 15.7 to 95.7% (mean 66.7% and SD 25.0%) in quiet and 0 to 87.1% (mean 50.4% and SD 30.7%) in noise. The wide variation in speech performance in the CI group is comparable with other data from international, large-scale research (Gifford et al., 2008; Blamey et al., 2013; Spahr et al., 2014), suggesting that the CI outcomes reported in the current study may be taken into account as representative of the general CI population. We considered those CI participants with word scores in quiet ≥88% and ≤ 50% (the top 11 and bottom 11 children from our cohort) to have good perception (good CI recipients, GCI) and poor speech perception (poor CI recipients, PCI), respectively. To avoid floor effects, the scores in quiet were selected for subsequent correlation analyses.

### 3.2. fNIRS results

#### 3.2.1. Data pre-processing

Some unacceptable channels were removed after the fNIRS data pre-processing steps, which included the exclusion of channels with poor signal quality using the SCI method and the application of motion artifact correction. In CI group, a total of 150 channels out of 1,672 channels (9.0%) met the exclusion criteria and were thus excluded from further analysis. Of these, 39 out of 570 (6.8%) available ROI channels were unusable. In NH group, 120 of 1,584 channels (7.6%) were excluded for further analysis. Of these, 40 out of 540 (7.4%) available ROI channels were unusable.

#### 3.2.2. Contrasts against silence

**Figure 2:**
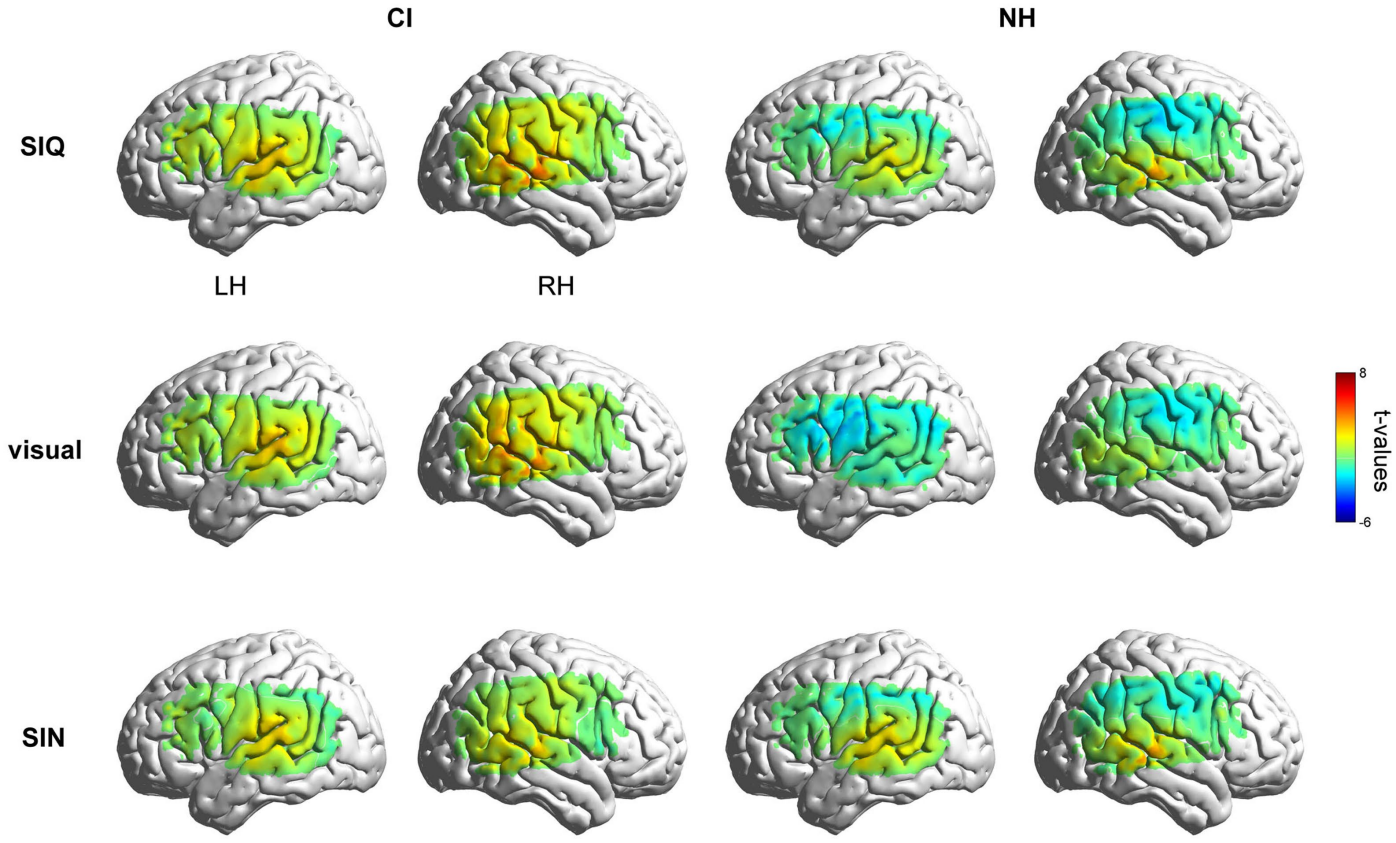
Group-level activation maps for CI and NH participants. Grand mean responses to each stimulus type were initially contrasted against the silence in the LH and RH, respectively. Note that the maps are interpolated from single-channel results, and the overlay on the cortical surface is for illustrative purposes only.

Figure 2 displays group-level activation maps for each condition compared to silence, for both groups. In the initial analysis, responses to stimuli were contrasted to the silent baseline, and tests were conducted on every individual fNIRS measurement channel. The NH group showed statistically significant activation (*q* < 0.05, FDR corrected) within channels overlying the right temporal gyri (Ch#38, 42) in SIQ and within channels overlying the left (Ch#16) and right (Ch#38, 42) temporal gyri in SIN. As expected, this group did not show any activation when responding to visual stimuli. The CI group showed larger activation in SIQ and the visual condition. Specially, statistically significant activation (*q* < 0.05, FDR corrected) was observed in channels overlying the left (Ch#12, 16) and right (Ch#33, 37, 38, 42) temporal gyri in SIQ, in channels overlying the right (Ch#38) temporal gyrus in SIN, and in channels overlying the left (Ch#12) and right (Ch#33, 37, 38, 42) temporal gyri in the visual condition. Additionally, during the processing of SIQ, CI children exhibited significant activation beyond the temporal cortex, localizing over LIFG (Ch#14). We used the mean values across the ROI measurement channels for subsequent analyses, as previous research on the reliability of fNIRS test–retest has consistently shown that averaging fNIRS response amplitude across a small number of channels located overlying a cortical ROI is more reliable than assessing it on a single-channel basis (Wiggins et al., 2016). Additionally, although there was no significant activation or deactivation within channels overlying IPL in both groups of children, we analyzed the cortical activation differences in IPL considering its potential to enhance speech recognition in challenging listening situations and the near-significant deactivation seen in NH group.

#### 3.2.3. ROI statistical analyses in the NH and CI groups

**Figure 3:**
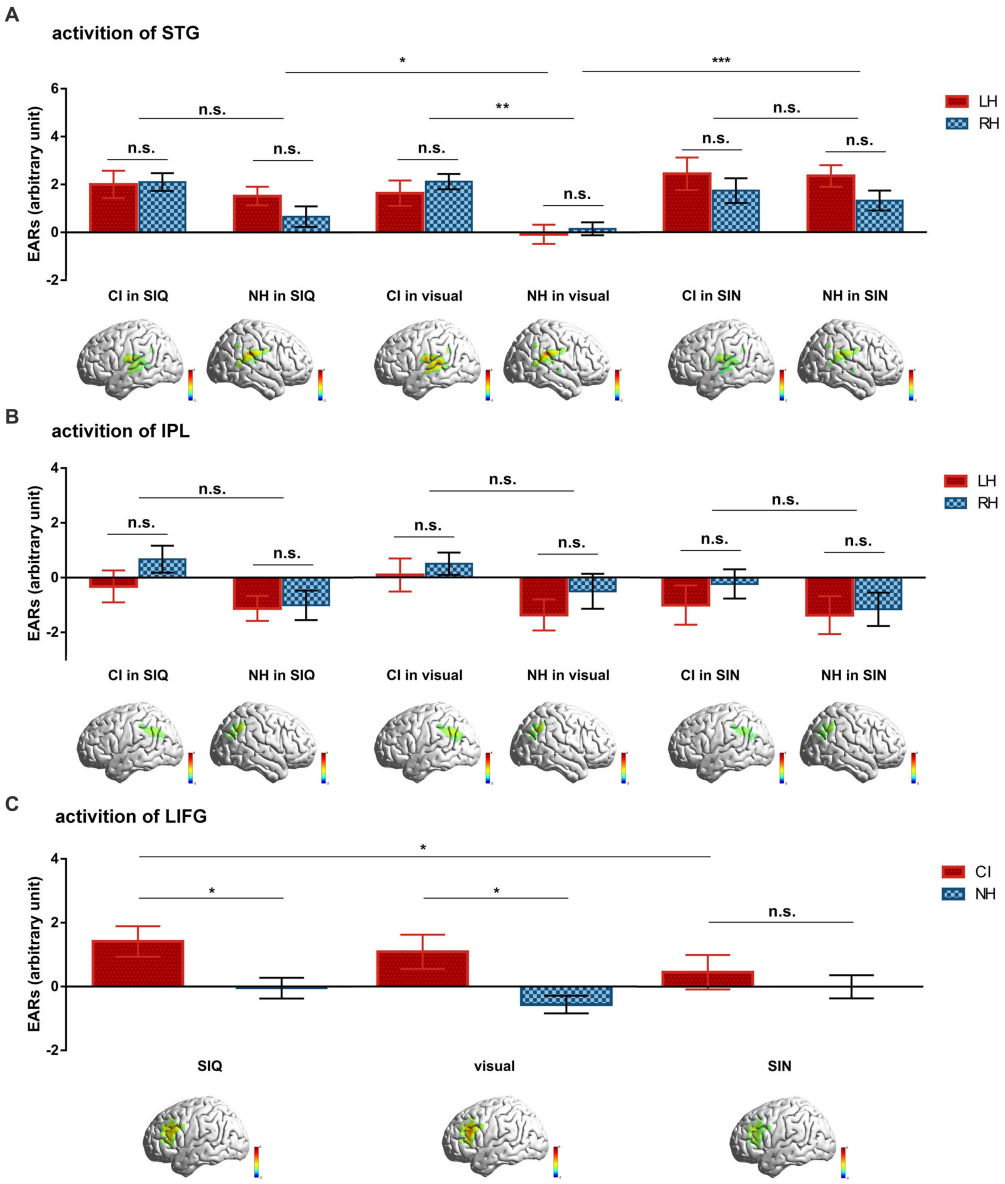
Differences in group-averaged ERAs for each ROI across all stimulus conditions between CI users and NH controls. **(A)** Cortical activation of STG. **(B)** Cortical activation of IPL. **(C)** Cortical activation of LIFG. Inset images below the statistics illustrate the differences in cortical activation maps for each ROI to corresponding stimulus between the two groups. Error bars represent standard error of the mean. Significance is marked as follows: *p < 0.05; **p < 0.01; ***p < 0.001; n.s., not significant.

To identify and address the experimental hypotheses, we used LMMs to compare differences in cortical activation for each ROI across all stimulus conditions between CI users and NH controls. The mean group-level ERAs for each ROI among conditions in both groups are shown in Figure 3.

To investigate cortical responses within STG, ERAs from the LH and RH were obtained from each participant for each condition (Figure 3A). These ERAs were then analyzed using a LMM with fixed effects of “group” (CI vs. NH), “stimulus type” (SIQ vs. SIN vs. visual condition), and “hemisphere” (LH vs. RH), along with all possible two- and three-way interactions. Furthermore, a random intercept for “participant” was included in the model. The results demonstrated that (i) there was a significant main effect of group (*F*(1,72) = 4.882, *p* = 0.030) and stimulus type (*F*(2,360) = 7.447, *p* = 0.001), (ii) there was a significant interaction between group and stimulus type (*F*(2,360) = 4.604, *p* = 0.011). Follow-up analyses for the group×stimulus type showed that (i) there was a significant difference in cortical responses to visual stimuli between CI users and NH subjects (*p* = 0.001), (ii) there were similar cortical response patterns between CI and NH participants for both levels of auditory stimuli (all *p* > 0.05), (iii) NH participants exhibited lower cortical activation in response to visual stimuli compared to SIQ (*p* = 0.017) or SIN (*p* < 0.001), and (iv) CI children displayed similar cortical responses across all conditions (all *p* > 0.05).

A second LMM was employed to examine cortical activation within IPL using the same parameter settings as in the STG analysis (Figure 3B). The results showed a significant main effect of hemisphere (*F*(1,360) = 6.205, *p* = 0.013); however, no significant interactions were observed between group and stimulus type or group and hemisphere (all *p* > 0.05).

A third LMM was used to investigate cortical activation in LIFG, with fixed effects of “group,” “stimulus type,” and “group×stimulus type,” along with a random intercept for “participant” (Figure 3C). Significant effects were observed for group (*F*(1,72) = 4.506, *p* = 0.037) and the group×stimulus type interaction (*F*(2,144) = 3.357, *p* = 0.038). The *post hoc* analyses for the group×stimulus type interaction revealed that (i) there were significant differences between CI users and NH participants in their cortical responses to SIQ (*p* = 0.022) and visual stimuli (*p* = 0.01), (ii) CI children exhibited lower cortical activation in response to SIN compared to SIQ (*p* = 0.019), and (iii) NH participants displayed similar cortical responses across all conditions (all *p* > 0.05).

#### 3.2.4. ROI statistical analyses within the CI group

**Figure 4:**
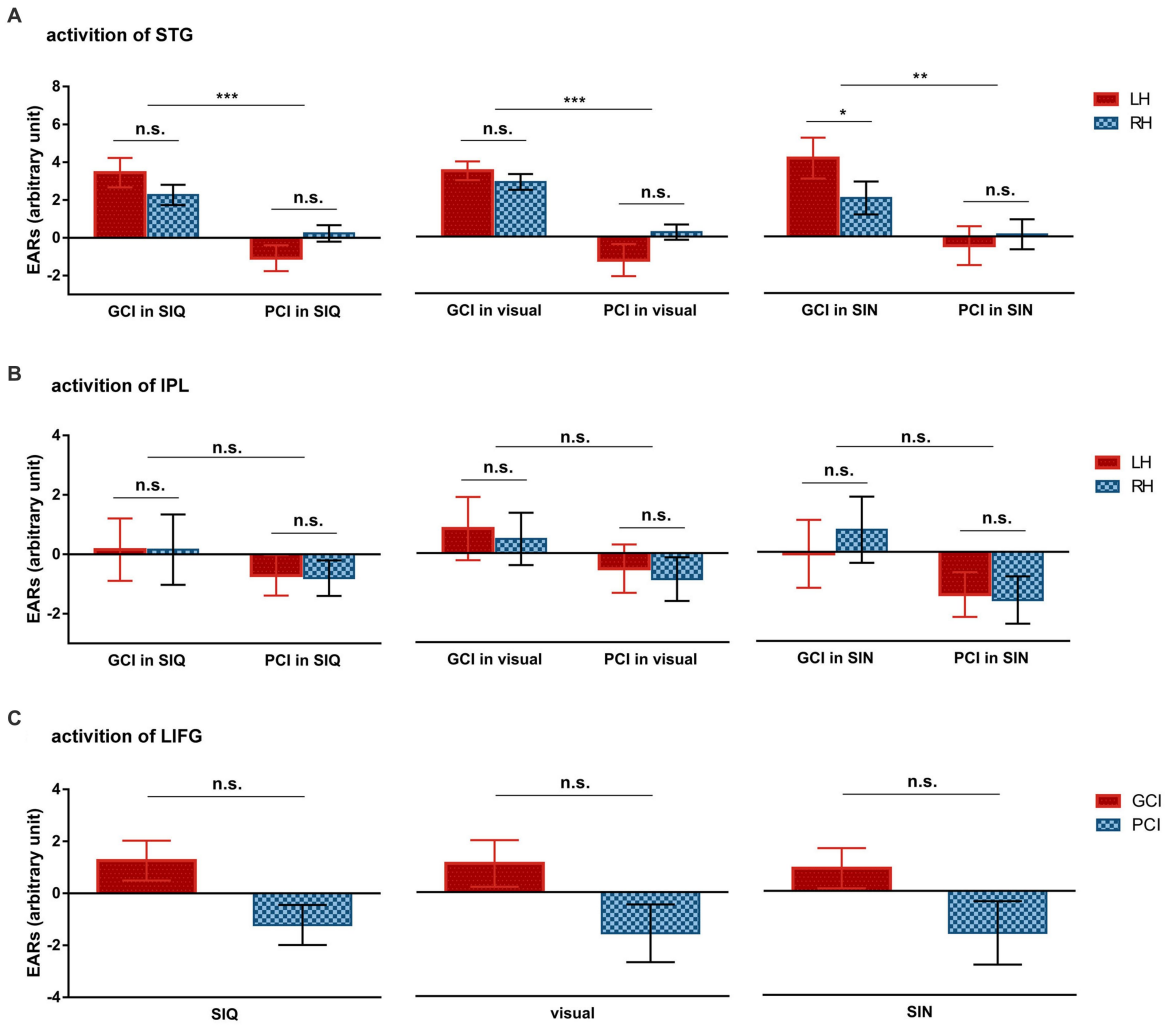
Differences in group-averaged ERAs for each ROI across all stimulus conditions between GCIs and PCIs. **(A)** Cortical activation of STG. **(B)** Cortical activation of IPL. **(C)** Cortical activation of LIFG. Error bars represent standard error of the mean. Significance is marked as follows: *p < 0.05; **p < 0.01; ***p < 0.001; n.s., not significant.

Given the huge variability in behavioral test scores among CI users, we conducted formal statistical analyses to compare cortical responses between GCIs and PCIs. We used the same statistical methods as previously described in Part 3.2.3, employing three LMMs to investigate differences in cortical activation between these two groups. The group-level means of ERAs for each ROI across conditions in both groups are depicted in Figure 4.

The LMM results for the STG revealed a statistically significant main effect of group (*F*(1,20) = 29.645, *p* < 0.001) and a significant interaction between group and hemisphere (*F*(1,100) = 10.779, *p* = 0.001). The *post hoc* analyses showed that GCIs exhibited greater activation than PCIs across all types of stimuli, including SIQ (*p* < 0.001), SIN (*p* = 0.009), and visual stimuli (*p* < 0.001). Additionally, the GCI group demonstrated significant LH dominance in SIN (*p* = 0.022), while there was no significant difference in activation between LH and RH in the PCI group across all speech conditions (all *p* > 0.05). The LMM results for the IPL found no significant effects (all *p* > 0.05).

The LMM results for the channels covering LIFG showed a statistically significant main effect of group (*F*(1,20) = 4.568, *p* = 0.045), but no significant interaction occurred between group and stimulus type (*F*(2,40) = 0.027, *p* = 0.974).

#### 3.2.5. Correlations with speech performance

**Figure 5:**
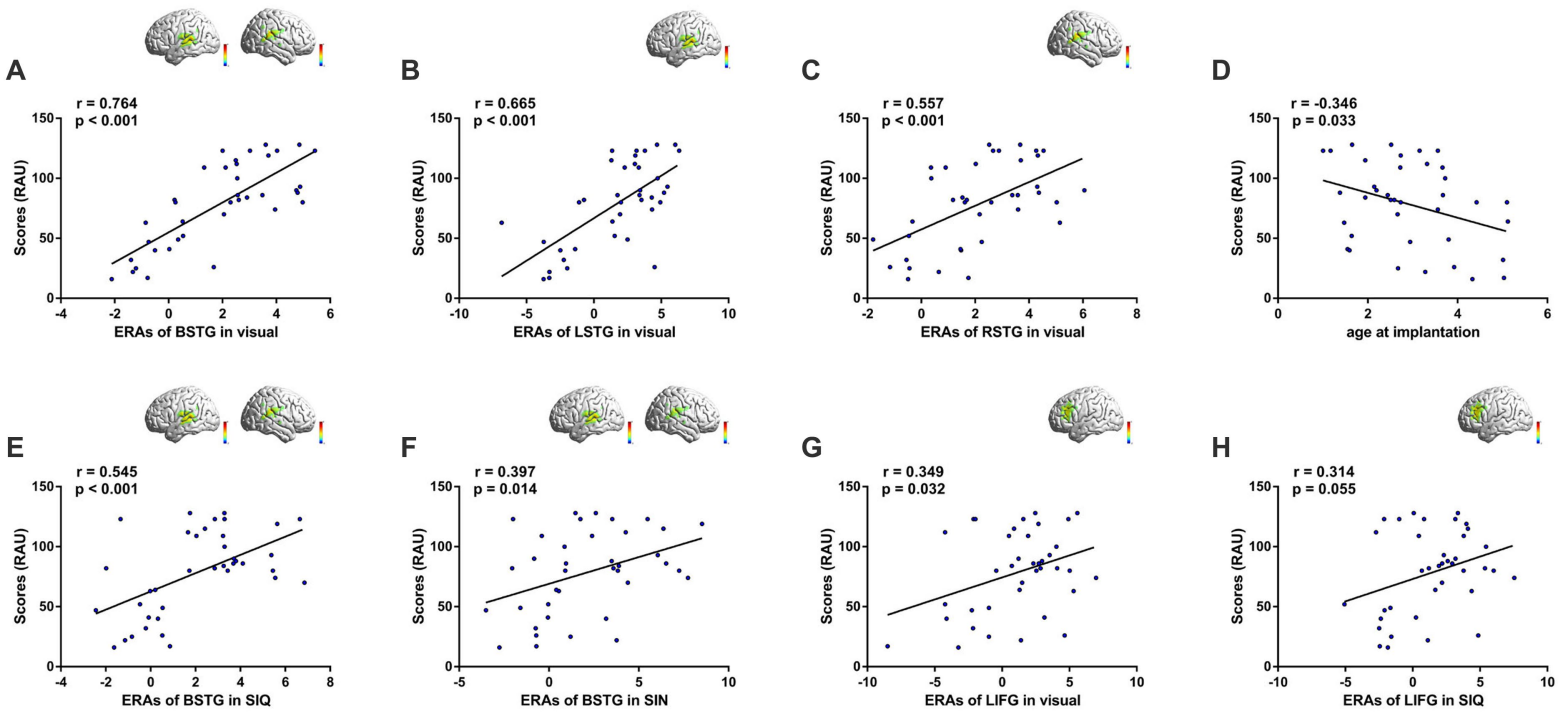
Cortical activation correlates with behavioral measures of speech perception. Inset images on the top right illustrate activation maps of the corresponding ROIs. **(A)** Correlation based on responses of bilateral STG (BSTG) to visual speech. **(B)** Correlation based on responses of LSTG to visual speech. **(C)** Correlation based on responses of RSTG to visual speech. **(D)** Correlation between age at implantation and CI performance. **(E)** Correlation based on responses of BSTG to SIQ. **(F)** Correlation based on responses of BSTG to SIN. **(G)** Correlation based on responses of LIFG to visual speech. **(H)** Correlation based on responses of LIFG to SIQ.

We conducted Pearson correlation analyses between speech performance in quiet (RAU) and ERAs in each ROI (Figure 5). There was a positive correlation between speech understanding and bilateral STG (BSTG) activation to visual speech stimuli (*r* = 0.764, *p* < 0.001; Figure 5A). To investigate whether this association was hemisphere-specific, separate correlation analyses were conducted for left (LSTG) and right (RSTG) regions, which showed that both hemispheres contributed to the relationship (LSTG: *r* = 0.665, *p* < 0.001; RSTG: *r* = 0.557, *p* < 0.001; Figures 5B,C, respectively). This finding suggested greater cross-modal visual responsiveness in STG among GCIs compared to PCIs. Although age-at-onset, duration of deafness, age-at-implantation and duration of CI use are common factors influencing CI outcomes, only age-at-implantation exhibited a negative correlation with CI outcomes (*r* = −0.346, *p* = 0.033; Figure 5D); no factors were correlated with temporal activation by visual speech (all *p* > 0.05). Even when controlling for age-at-implantation using partial correlation analysis, a strong positive correlation between cross-modal activation and speech understanding remained (*r* = 0.750, *p* < 0.001). Furthermore, low-to-moderate correlations were found between CI outcomes and bilateral STG activation to SIQ (*r* = 0.545, *p* < 0.001; Figure 5E) and SIN (*r* = 0.397, *p* = 0.014; Figure 5F).

Interestingly, we found activation in LIFG in response to visual speech stimuli to be weakly correlated with CI outcomes (*r* = 0.349, *p* = 0.032; Figure 5G). Furthermore, we observed a nearly significant correlation between LIFG activation in response to SIQ and speech performance (*r* = 0.314, *p* = 0.055; Figure 5H). In contrast, we did not find any significant association between cortical responses in IPL and CI performance.

Overall, we believe that the results of the correlation analysis were largely consistent with those of the activation analysis, although only activation within STG in response to visual speech and SIQ remained significantly correlated with speech test scores for CI users when using the Bonferroni correction to reduce the possibility of type I errors during a series of correlation analyses. The absence of any noteworthy correlation between cortical responses in IPL and CI outcomes may be due to the unclear impacts of neural activity of IPL in this study. The activation response patterns in IPL were considerably disparate, even for GCIs, comprising both deactivation and activation responses. In the future, it will be necessary to expand the sample size and explore the effects of speech recognition accuracy on cortical activation in the parietal cortex in CI users further.

#### 3.2.6. Functional connectivity: statistical analyses between NH and CI groups

**Figure 6:**
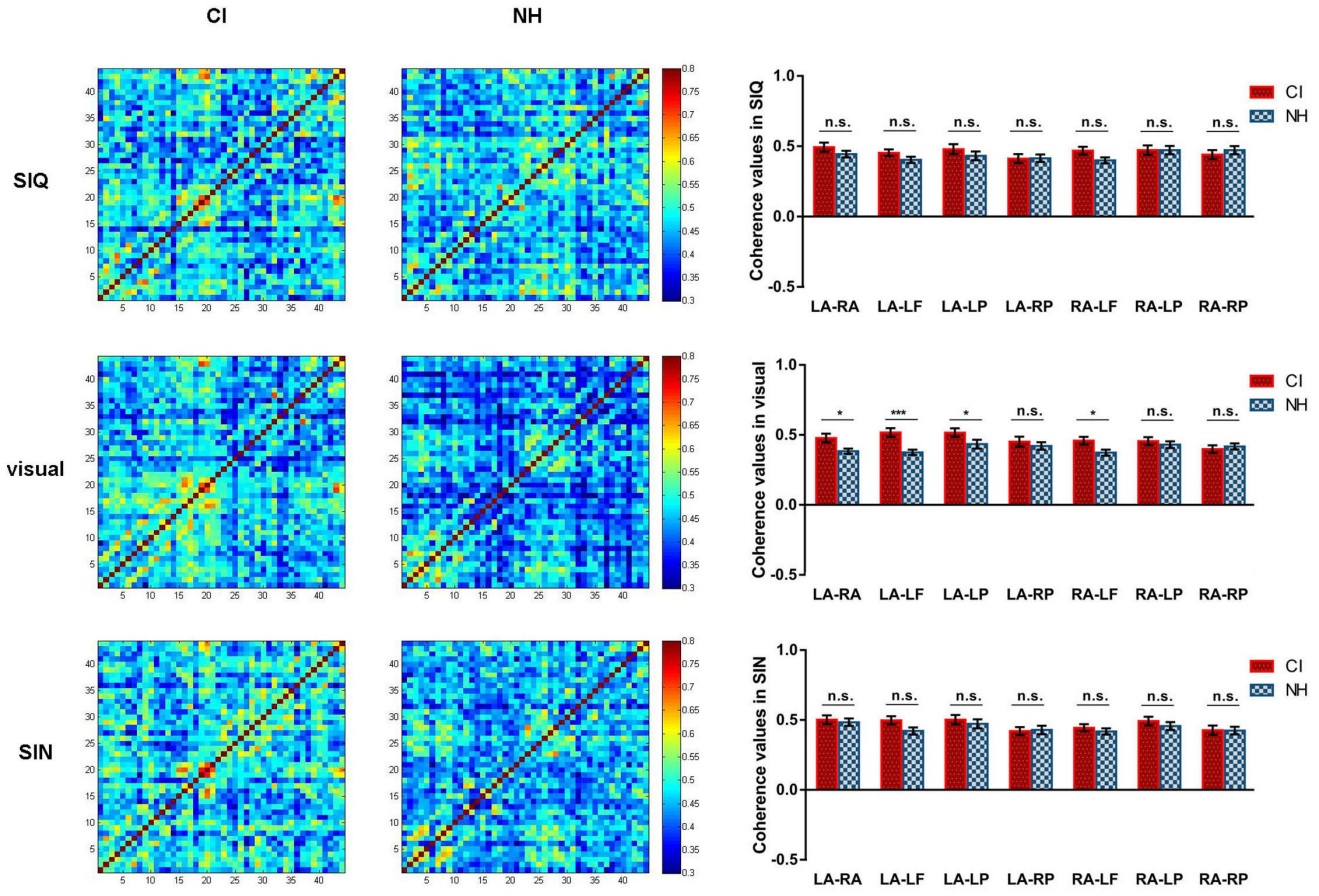
Differences in functional connectivity across all stimulus conditions between CI group and NH group. For each stimulus condition, the group-averaged coherence values of measuring channels are plotted separately for CI children (left column) and NH participants (middle column). The rightmost column shows the differences in coherence values for each ROI pair between the CI and NH groups. LA, LSTG; RA, RSTG; LF, LIFG; LP, LIPL; RP, RIPL. Error bars represent standard error of the mean. Significance is marked as follows: *p < 0.05; ***p < 0.001; n.s., not significant.

After demonstrating activation differences between CI children and NH controls, we further explored possible mechanisms by analyzing task-related functional connectivity between 7 pairs of ROI in response to visual and two levels of auditory speech stimuli within these two groups. Figure 6 displays the results of functional connectivity analysis for the CI and NH groups, respectively.

The LMM results for visual speech stimuli indicated a significant main effect of group (*F*(1,72) = 7.701, *p* = 0.007) and group×ROI pair interaction (*F*(6,432) = 2.346, *p* = 0.031). Further analysis of the group×ROI pair interaction revealed that the task-related functional connectivity differed significantly between the NH and CI groups in various ROI pairs, including LSTG and RSTG (*p* = 0.018), LSTG and LIFG (*p* < 0.001), LSTG and LIPL (*p* = 0.037) and RSTG and LIFG (*p* = 0.031), with stronger connectivity observed in CI children as compared to those with NH.

The LMM results for SIN revealed only a significant main effect of ROI pair (*F*(6,432) = 3.172, *p* = 0.005). In contrast, there were no statistically significant effects with respect to main effect of group or group×ROI pair interaction (all *p* > 0.05). Similarly, in terms of responses to SIQ, no statistically significant effects were found either (all *p* > 0.05).

To investigate the relationship between task-related functional connectivity and speech recognition ability, Pearson correlation analyses were performed for speech performance in quiet (RAU) and coherence values for each ROI pair in response to each stimulus type. However, no significant associations were found between task-related functional connectivity and CI outcomes (all *p* > 0.05).

## 4. Discussion

This study used fNIRS to investigate brain activation in pre-lingually deaf CI children to three types of speech stimulus and their correlations, especially visual cross-modal activation in STG, with behavioral speech perception after implantation. We aimed to extend previous findings to more representative CI children with pre-lingual deafness and to larger cortical regions with regard to speech understanding and effortful listening. The findings indicate that cortical responses of STG in CI children, especially those GCIs, were on average greater than those in NH group when processing all speech stimuli. Additionally, activation of STG was significantly correlated with behavioral speech test scores in quiet, with strong positive correlations observed between cross-modal activation within STG and CI performance. Specifically, better speech comprehension with a CI was associated with stronger STG activation in response to visual speech. A secondary analysis revealed that CI children, particularly GCIs, exhibited increased responses to all experimental speech stimuli in the LIFG region compared to NH controls. Additionally, there was a nearly significant correlation between LIFG activation in response to SIQ or visual speech and CI outcomes. The results suggest that visual cross-modal reorganization is at least one of the neural bases of poor speech perception in CI participants and that cortical activation of the LIFG may be a cortical marker for effortful listening. As far as we know, this is the first fNIRS research to describe neural activation of functional fronto-temporal–parietal networks involvement in speech comprehension and cross-modal reorganization in pre-lingually deafen CI children with a diverse range of speech abilities.

### 4.1. Cross-modal responses of auditory regions in CI users and in NH controls

The observation of significantly higher visual-evoked activation of auditory cortex in deaf CI users compared with NH controls aligns well with previously published data (Finney et al., 2001, 2003; Karns et al., 2012; Vachon et al., 2013). It remains a subject of debate regarding how such cross-modal reorganization of temporal regions may impact hearing restoration in pre-lingually deaf children after implantation. In this study, we involved pre-lingually deaf CI children with a more diverse range of speech abilities to study the relationship between cross-modal activation by visual speech stimuli and speech performance with CIs. To our knowledge, the sample size of this study, consisting of *n* = 38 CI participants, is the largest in this field and this increased sample size was expected to increase statistical power. It is interesting to note that our data did not support the theory that responsiveness of bilateral STG to visual speech was negatively correlated with CI success, but instead suggested that greater recruitment of auditory brain regions for processing visual speech would facilitate the restoration of hearing after implantation. Specially, participants with well-performing CIs achieved a greater cross-modal response than those with poorly performing CIs. Additionally, this positive relationship was not driven predominantly by one cerebral hemisphere. We also demonstrated that early implantation was closely related to better speech outcomes. However, this relationship seems not to be done by preventing cross-modal reorganization because there was no correlation between age-at-implantation and cross-modal activation. Perhaps one of the reasons is that early implantation contributes to the “normal” development of the auditory pathway during the sensitive period for auditory processing, or greater implantation age is linked to reduced gains from audiovisual integration (Stevenson et al., 2017). Another possibility is that there may be an undisclosed correlation between these two as the study did not examine cortical activation levels in deaf children before implantation. A more reasonable approach to identifying this correlation would be to investigate cross-modal responses to visual speech preimplantation or to measure cortical changes from deafness to hearing recovery.

Recent research utilizing fNIRS have reported a comparable association between visual-evoked activation in the auditory cortex and speech perception with CI (Anderson et al., 2017; Mushtaq et al., 2020). In a longitudinal fNIRS study, Anderson et al. (2017) reported that enhanced visual cross-modal activation among individuals with CI correlated with better auditory speech understanding ability following implantation. However, unlike in the current study, Anderson et al.’s investigation included pre-lingually, peri-lingually, and post-lingually deaf adults, and the speech understanding was tested in the best-aided condition, which included hearing aids for many participants. Therefore, it remains unclear whether this association was driven by group disparities or residual hearing (Zhou et al., 2018). Mushtaq et al. (2020) subsequently investigated the activation of temporal cortex to visual and auditory speech stimuli in pre-lingually deaf CI children. The study confirmed that visual cross-modal plasticity provides adaptive benefits for restoring hearing with CI through an audiovisual mechanism. However, it remains uncertain whether the better speech skills in some pediatric CI users result from an innate ability to combine visual information with auditory input from birth or develop over time and with experience in those who already have good listening skills with CI.

Our findings fill in the gaps in this field and contribute to the existing evidence that a stronger visual processing ability in the auditory areas is positively related to successful CI outcomes (Jean-Luc et al., 2004; Strelnikov et al., 2013, 2015; Anderson et al., 2017; Mushtaq et al., 2020). Our study suggests that visual cross-modal reorganization was at least one of the neural bases of variable speech perception in pre-lingually deaf CI participants. Several potential reasons and mechanisms have been proposed to interpret the facilitative link between visual takeover of auditory brain regions and auditory speech understanding with CI. One possibility is an increase of the direct anatomical connection between visual and auditory cortical areas (Bizley et al., 2007; Chen et al., 2016) or a highly inherent correspondence between auditory and visual speech representations (Anderson et al., 2017). This supports the notion that CI users might become better at integrating auditory and visual speech cues as a compensatory mechanism (Mushtaq et al., 2020). Another proposal is that that vision may facilitate auditory perceptual learning by guiding top-down attention to auditory representations (Bernstein et al., 2013) or by assisting to decipher the degraded auditory speech when the incoming auditory signal is insufficient or in challenging listening environments (Strelnikov et al., 2009). Thirdly, it has been argued that the sensitive period for auditory processing should be viewed concurrently with the sensitive period for language processing (Lyness et al., 2013). Therefore, visual take-over of the auditory cortex after hearing deprivation could promote the development of language function in the critical period, which may be beneficial to the prognosis following CI (Lyness et al., 2013).

### 4.2. Intra-modal responses in CI users and in NH controls

We found that CI users processed auditory input similarly to NH children. Interestingly, further analysis revealed there was a stronger activation of STG in GCIs and a lower activation in PCIs, when compared to NH group. This is a little different from our experimental hypothesis and previous study (Olds et al., 2016) that the response of GCI should be similar to that of NH listeners to demonstrate “normal.” The reason may be that GCIs required more neural activity to accurately decode degraded speech signals coded by a neuroprosthetic device than NH listeners did to decode natural speech signals (Yasushi et al., 2000; Mushtaq et al., 2020), while most PCIs may judge the process of decoding too difficult to succeed, resulting in decreased activity in auditory brain regions. Alternatively, perhaps the difference between pre-lingual and post-lingual deafness, or some other unknown factors led to this result. In any case, more research in this area is required to confirm this.

Our finding of a non-significant increase in STG responses to SIN compared to SIQ in both GCI and NH groups is consistent with the idea that greater neural activity in auditory regions was required in noise vs. quiet to maintain the same speech performance (Lawrence et al., 2021). However, for age-matched NH listeners, there was almost no difference between the noise condition of +10 dB SNR and the quiet condition, because the SRT of NH individuals is often lower than 0 dB SNR according to previous research (Chen et al., 2020). Furthermore, the +10 dB SNR condition was designed with high intelligibility where ceiling performance was presented in an adult fNIRS study (Defenderfer et al., 2021). This may also be the reason why the activation amplitude of STG or LIFG showed no significant distinction between these two conditions in the control group. On the contrary, in the cases of CI children, especially PCIs, both of the two auditory conditions were not easy for them, making them differ modestly in average score. We could infer that the lack of a significant difference in STG activation between the auditory conditions was due to the combined effects of lower speech recognition scores and higher neural activity under the noise condition in comparison to that under the quiet condition. Additionally, it is suggested that the intensity of the stimulus and the perception of the stimulus can play an important role in respect to the activation amplitude (Weder et al., 2018, 2020). Future work should also focus on identifying the mechanisms of brain activation by speech sounds with varying SNR.

While there were no significant differences in STG activation between LH and RH in either group, both the NH and GCI groups exhibited a tend of left hemisphere dominance when processing two levels of auditory stimuli. Additionally, a significant hemispheric lateralization was seen in the GCI group during their response to SIN. In contrast, PCIs did not show this similar left-hemispheric dominance for activation in STG. This seems supporting the finding of left-hemispheric dominance for language processing (Lazard et al., 2012; Paquette et al., 2015). Interestingly, a low-to-moderately positive correlation was demonstrated between between speech perception scores in CI children and STG responses to both SIQ and SIN, which implies that the STG is critical for auditory stimulus encoding and processing, as well as correlating with speech intelligibility (Pollonini et al., 2014; Olds et al., 2016; Lawrence et al., 2018; Mushtaq et al., 2021).

### 4.3. Potential cortical correlates of effortful listening

#### 4.3.1. The role of LIFG In effortful listening

Previous research has identified frontal and pre-frontal cortical involvement in the processing of visual information in hearing loss (Rosemann and Thiel, 2018; Glick and Sharma, 2020). The current study also suggests that the LIFG showed significantly greater responses to visual sentences in the GCI group than those in the PCI and NH groups. The increased levels of LIFG activation to visual speech in GCIs might be due to a top-down mechanism to modulate visual cross-modal reorganization and speech perception outcomes. Similarly, PCIs and NH controls showed deactivation of LIFG in this condition, consistent with a lack of cross-modal reorganization. Alternatively, there may be a stronger task-related functional connection between LIFG and the auditory or visual regions in GCIs. Our data seem to support a prior finding from a PET study, which suggested that the deaf children who had developed greater executive and visuospatial functions subserved by the prefrontal cortex might be successful in auditory language learning after CI (Lee et al., 2005).

Additionally, we observed an obvious activation of the LIFG among GCIs when presented with SIQ and SIN stimuli, whereas the control group only showed slight LIFG activation in response to the SIN. The PCI group did not exhibit any LIFG activation in response to either the SIQ or SIN stimuli. As mentioned before, LIFG has been identified as one brain region potentially involved in effortful listening (Wong et al., 2008; Obleser and Kotz, 2010). This region supports the recovery of meaning from degraded speech or acoustically challenging speech by a greater level of top-down cognitive processing. The phenomenon is confirmed both in NH listeners (Sohoglu et al., 2012) and in hearing-impaired population with CIs (Sherafati et al., 2022). In our study, we chose an SNR of 10 dB, one reason is to correspond with the noise condition of behavioral test, and the other reason is that the average score of CI children was 50.6% in this condition, which was almost equal to SRT (defined as the SNR that produced 50% correct word recognition). However, the difficulty of speech recognition in the noise condition of +10 dB SNR for NH listeners was similar to that in a quiet environment, because speech scores in the two conditions were almost perfect. Previous studies have demonstrated that the SRT of NH individuals was far below +10 dB (the lowest SRT is −22.9 dB) (Chen et al., 2020) and speech in the +10 dB SNR condition was high intelligibility, with ceiling performance observed in NH adults (Defenderfer et al., 2021). This may be the reason why LIFG was not significantly activated in the control group under both the quiet and noisy conditions, or why no difference in LIFG activation was found in the control group between the two auditory conditions. Conversely, in cases of GCIs, greater activation of LIFG was possibly associated with more listening effort since they have to utilize more cognitive resources to effectively discriminate speech signals. Additionally, the slightly higher ERAs of LIFG to SIQ compared to the SIN in these children may be due to either suboptimal behavioral performance in quiet or the immature function of LIFG. The deactivation of LIFG for PCIs suggested that these individuals may identify the experimental trials as impossible and eventually “gave up” (Pichora-Fuller et al., 2016), like the response of the STG. Briefly, our results confirmed that the increase in LIFG brain activation may be a cortical marker for effortful listening, at least for CI children. Future work needs to set more different levels of SNR to validate the role of LIFG in recognizing degraded speech in children with CI and NH.

#### 4.3.2. The role of IPL in effortful listening

Our data suggests that there were no significant differences in activation of IPL under all speech conditions between the CI and NH groups, or between the GCI and PCI groups. However, a global deactivation of this region was observed in response to each type of speech stimuli in both PCIs and NH participants. Conversely, in GCIs, we observed a global activation except for the LIPL response to SIN. It has been suggested that inferior parietal regions are part of the default mode network (DMN), which are preferentially more active during “rest” vs. engagement in an external task (Buckner et al., 2008), and the strength of deactivation within the DMN has been shown to correlate with task difficulty (Wild et al., 2012; Lawrence et al., 2018). Thus, we initially hypothesized that the level of deactivation may be greatest in CI group, particularly in GCI subgroup, similar to the activation trend of LIFG. Unexpectedly, the response patterns seemed to be completely different from what we expected. Indeed, IPL, beyond its role as an area of the DMN, is also known to be extensively involved in facilitating comprehension through the use of linguistic and semantic context (Obleser and Kotz, 2010; Golestani et al., 2013; Hartwigsen et al., 2015) and to form part of a functional fronto-temporal–parietal network supporting speech comprehension (Abrams et al., 2013). For instance, increased neural activity in IPL, especially in the angular gyrus of the left IPL, accompanies successful comprehension in challenging listening conditions (Bonner et al., 2013; Erb et al., 2013; Golestani et al., 2013; Clos et al., 2014). The precise role of bilateral IPL in this study is unknown. It seems likely that both deactivation of the DMN network and activation of the speech comprehension network may contribute to the response patterns in bilateral IPL in the current study because we could not explain the results using only one of networks. As such, further work is needed to clarify the role of IPL involvement in visual cross-modal reorganization and speech intelligibility among the hearing-impaired population.

### 4.4. Functional connectivity

We observed that CI children exhibited significantly higher task-related functional connectivity for visual stimuli than NH children in the main ROI pairs, particularly between the interhemispheric auditory cortex, between the auditory region and LIFG, as well as between the left auditory area and LIPL. This indicates that CI users rely on more networks than NH controls when processing visual sentences, which involve areas such as STG, LIFG, and LIPL. In a prior fNIRS study, Fullerton et al. (2022) examined cross-modal functional connectivity between auditory and visual cortices in a sample of post-lingually deaf CI adults and age-matched NH controls. They demonstrated that CI users had greater cross-modal functional connectivity between left auditory and visual cortices for speech stimuli, irrespective of the type of sensory modality, compared to NH controls, and that cross-modal functional connectivity for visual speech was positively correlated with CI outcomes. They thus concluded that CI adults with post-lingual deafness may be able to engage a distributed, multimodal speech network to improve speech understanding. Our research revealed enhanced task-related connectivity in response to visual stimuli when compared to NH participants, corroborating Fullerton et al.’s (2022) findings. This provides further evidence that CI users may have improved multisensory integration and more extensive neural networks for speech or language processing. Finally, this multimodal interaction reinforces our previous cortical activation analyses that showed increased responses in fronto-temporal–parietal regions, particularly superior cross-modal activation in temporal regions by visual speech among proficient CI children. Regrettably, the optode configuration of fNIRS did not include the visual cortex in our study, preventing us from analyzing different functional networks that involve visual brain regions. Perhaps there is no direct functional connection between auditory cortex and frontoparietal areas; instead, cortical activation and coherence values may reflect responses in another functional network, such as the connections between visual cortex and auditory regions or between visual cortex and the frontoparietal network In the future, it will be necessary to further explore the activity of different functional networks during speech processing in pre-lingually deaf CI children, which should include but are not limited to visual cortex, auditory cortex, and frontoparietal areas.

### 4.5. Potential applications in clinic

Restoring a deaf person’s ability to recognize and distinguish auditory speech is the primary objective of the surgical implantation of CI. As indicated before, a number of variables, including age-at-onset, duration of deafness prior to implantation, age-at-implantation, and duration of CI use, can affect speech outcomes in CI users (Tomblin et al., 2005; Lin et al., 2008; Niparko et al., 2010; Tobey et al., 2013). However, in our study, only age-at-implantation was negatively correlated with CI outcomes. This result supports the previous theory that such known variables can explain only a small portion of the variance in CI speech outcomes, leaving a considerable portion unexplained (Niparko et al., 2010; Geers et al., 2011). It is worth noting that the relationship between age-at-implantation and speech performance with a CI is weak (*r* = −0.346); therefore, it may be inaccurate to rely solely on this variable to predict speech outcomes following implantation. Our current findings in a group of pre-lingually deaf CI users suggest a strong correlation (*r* = 0.764) between cortical activation of STG in response to visual speech and speech understanding ability with a CI, even after controlling the confounding variables. Additionally, cortical activation of the LIFG could serve as a potential cortical marker for effortful listening in CI children. In summary, fNIRS-based measurements of cortical activation, particularly the cross-modal responses of STG, may provide objective, additional value to help with a more precise prognosis of CI outcomes. Furthermore, using these neuromarkers in combination with behavioral speech understanding tests is also more beneficial and efficient to guide post-implant programming, modify rehabilitation training strategies, and assess speech performance, especially for infants and children.

### 4.6. Limitations

One limitation is that although comparable speech materials were used in both the behavioral speech understanding test phase and the neuroimaging phase to avoid training effects, our inference of trial accuracy in the neuroimaging phase based on the behavioral results is not accurate enough. In addition, our paradigm does not allow us to differentiate brain activation between correct trials and incorrect trials or to investigate the correlation between the levels of cortical activation and response time. Future studies should explore the speech recognition accuracy in the neuroimaging phase and its effects on cortical activation in the temporal, frontal, and parietal cortex of individuals with hearing loss, both with and without hearing devices. Another noteworthy limitation is that the optode configuration of fNIRS used in our study did not include the visual cortex, preventing us from examining the functional connection between visual regions and auditory regions or other brain regions. There are also some limitations to using fNIRS as a diagnostic tool, despite its positive attributes, as discussed in the previous paragraph. One major drawback is that fNIRS can only image superficial regions of cortex in humans due to its shallow imaging depth. Furthermore, scalp thickness may interfere with the ability of fNIRS to accurately image cortical activity. Additionally, not all participants are able to tolerate the discomfort or tightness caused by the fixation of optodes, making fNIRS imaging impossible in some cases.

## 5. Conclusion

In conclusion, the current fNIRS study revealed that: (1) compared to PCIs or NH controls, the temporal regions exhibited significantly greater activity to visual speech in GCI group; (2) an increase in activation of auditory brain regions to both auditory and visual speech in CI users were directly correlated to auditory speech understanding ability, with the strongest positive association between cross-modal brain plasticity and CI outcome; (3) beyond STG, brain activation of LIFG would be associated with a top-down modulatory mechanism to visual cross-modal reorganization and recovery of meaning from degraded speech; (4) the precise role of neural activity in inferior parietal regions was unclear, perhaps referring to both deactivation of the DMN and activation of the speech comprehension network. We suggest that cross-modal reorganization in auditory cortices may be at least one of the neural bases of highly variable CI performance due to its beneficial effects for speech understanding, thus supporting the ability to predict and assess CI prognosis, and that cortical activation of the LIFG may be a cortical marker for effortful listening. According to our research, fNIRS can identify functional brain differences between CI users and NH listeners that are associated with their auditory speech understanding following implantation. As a result, fNIRS may have the potential to be used in the clinical management of CI candidates and users, either in evaluating speech intelligibility objectively at the cortical level or in directing rehabilitation strategies.

## Data availability statement

The raw data supporting the conclusions of this article will be made available by the authors, without undue reservation.

## Ethics statement

The studies involving human participants were reviewed and approved by the Ethical Committee of Chongqing General Hospital. Written informed consent to participate in this study was provided by the participants’ legal guardian/next of kin. Written informed consent was obtained from the individual(s), and minor(s)’ legal guardian/next of kin, for the publication of any potentially identifiable images or data included in this article.

## Author contributions

X-QZ: experimental design, fNIRS paradigm writing, data collection, data processing, and manuscript writing. Q-LZ: neuroimaging data collection. XX: fNIRS test materials provision and fNIRS stimuli editing. M-RL, HL, SL, and TZ: behavioral data collection. WY: experimental design, project implementation management, and manuscript review. All authors contributed to the article and approved the submitted version.

## Funding

This work was funded by Chongqing Municipal Public Health Bureau, Chongqing People’s Municipal Government (No. 2022DBXM006), National Natural Science Foundation of China (No. 81873702), and Chongqing Science and Technology Commission (Nos. 2022NSCQ-MSX2839 and CSTB2022NSCQ-MSX0553).

## Conflict of interest

The authors declare that the research was conducted in the absence of any commercial or financial relationships that could be construed as a potential conflict of interest.

## Publisher’s note

All claims expressed in this article are solely those of the authors and do not necessarily represent those of their affiliated organizations, or those of the publisher, the editors and the reviewers. Any product that may be evaluated in this article, or claim that may be made by its manufacturer, is not guaranteed or endorsed by the publisher.
